# Current and Emerging Technology for Continuous Glucose Monitoring

**DOI:** 10.3390/s17010182

**Published:** 2017-01-19

**Authors:** Cheng Chen, Xue-Ling Zhao, Zhan-Hong Li, Zhi-Gang Zhu, Shao-Hong Qian, Andrew J. Flewitt

**Affiliations:** 1 School of Environmental and Materials Engineering, College of Engineering, Shanghai Polytechnic University, Shanghai 201209, China; chencheng@sspu.edu.cn (C.C.); xlzhao@sspu.edu.cn (X.-L.Z.); zhli@sspu.edu.cn (Z.-H.L.); 2 Department of Ophthalmology, Eye and ENT Hospital, Shanghai Medical College, Fudan University, Shanghai 200231, China; qsh2304@163.com; 3 Electrical Engineering Division, Department of Engineering, University of Cambridge, J J Thomson Avenue, Cambridge CB3 0FA, UK; ajf@eng.cam.ac.uk

**Keywords:** continuous glucose monitoring, glucose biosensor, implanted devices, mini-invasive, non-invasive

## Abstract

Diabetes has become a leading cause of death worldwide. Although there is no cure for diabetes, blood glucose monitoring combined with appropriate medication can enhance treatment efficiency, alleviate the symptoms, as well as diminish the complications. For point-of-care purposes, continuous glucose monitoring (CGM) devices are considered to be the best candidates for diabetes therapy. This review focuses on current growth areas of CGM technologies, specifically focusing on subcutaneous implantable electrochemical glucose sensors. The superiority of CGM systems is introduced firstly, and then the strategies for fabrication of minimally-invasive and non-invasive CGM biosensors are discussed, respectively. Finally, we briefly outline the current status and future perspective for CGM systems.

## 1. Introduction

Diabetes mellitus is a worldwide epidemic disease affecting 422 million people, and is predicted to be the seventh leading cause of death if the current morbidity trends continue [1]. Blood glucose (BG) concentrations in diabetics can undulate significantly throughout a day, and they lead serious consequences including kidney failure, strokes, heart attacks, high blood pressure, blindness and coma [2,3]. BG levels could be finely controlled by insulin, and the abnormal concentration of BG is caused by the absence of insulin secretion (type I diabetes, T1D) or defective insulin secretion and action (type II diabetes, T2D). For maintaining BG within the euglycemic range, the BG concentration should be detected at least twice a day and four times a day for T2D patients and T1D patients, respectively, and combined therapies including drugs, exogenous insulin supply, diet and physical exercise [4,5,6]. The emergence of glucose sensors has provided patients the ability to self-monitor BG levels so as to manage insulin levels, and thus control the mortality of diabetes mellitus. Traditional glucose detecting devices primarily consist of glucose sensors based on electrochemical methods [7]. The frequent analysis of BG levels requires a small blood sample (<1 μL) obtained by a “finger-pricking” collection method, which is inconvenient and results in poor patient compliance. Such tests neglect nighttime variations and might cause approximation of BG variations. Moreover, instantaneous monitoring sensors cannot provide real-time BG information, and thus are unable to warn of hypoglycemic (low blood sugar, <3.0 mM) and hyperglycemic (high blood sugar, >11.1 mM) events in advance.

There are three generations in the development of glucose biosensors, the first-generation relied on the use of the natural oxygen and the production-detection process of hydrogen peroxide, and the second-generation sensors employ a non-physiological electron acceptor to shuttle electrons and thus solve the oxygen deficiency. The market leading methods are based on second generation sensor technology. The design of the third-generation glucose sensors aims to get rid of the leachable artificial mediators and even the glucose enzyme. Therefore, continuous glucose monitoring (CGM) devices are considered to be the ideal candidate for the next generation products to replace the currently used portable glucose meters [8]. CGM reports BG levels as trends of glucose fluctuations during a day (including the increasing or decreasing of BG), and these data could be utilized for various applications, including retrospective analysis [9], hypo/hyperglycemia detection and prediction [10,11]. Ideally, intelligent CGM devices could be linked to an insulin delivery pump to form an artificial pancreas [12], and the benefit of CGM point-of-care tests for the self-management of diabetes patients is the reduced time length spent in hypoglycemia and the increased time in euglycemia [13,14,15]. However, the current gold standard glucose biosensors are invasive, and CGM systems still present a number of limitations, such as biofouling, fibrous encapsulation of the implanted electrode, inflammation, and loss of host vasculature, which seriously affect the precision and accuracy of the BG results [16]. Non-invasive methods, on the other hand, are increasingly prevalent due to the highly sensitivity and better patient compliance contrary to invasive ones [17]. Since there is a delay as glucose is transported from the blood level to the interstitial fluid level, the real-time information detected from CGM sensors might be 15–20 min later than BG, so the lag-of-time of interstitial glucose (IG) relative to BG limits the result reliability in hypoglycemic emergency situations. Although, many enhancements have been implemented to mitigate the inaccuracy of CGM, there is still lack of approval of CGM data for adjusting insulin, since the dosage of insulin is strongly dependent on the BG level and rate of change of BG. Another barrier is the lifetime of the sensors, as loss of function happens to most implantable electrodes within 7 days, and the calibration would also create a series of problems such as cost, toxicity, inconvenience and discomfort [18]. The integration of CGM with wireless devices could be an effective method for developing closed loop systems like the Internet of Things. By 2017, there will be more mobile phones around world than people, and the CGM data could be sent to the smart phones for analysis to then trigger the release of drugs [19].

This review focuses particularly on the progress in the development of CGM technologies, focusing specifically on subcutaneous implantable electrochemical glucose sensors, which are widely studied and commercially available. We briefly introduce the superiority of CGM systems and discuss the challenges in the development and implementation of CGM devices in Section 1. Strategies for the fabrication of minimally-invasive CGM biosensors, including new materials, biocompatible coatings and drug delivery systems, are presented in Section 2. The non-invasive technologies and concepts are introduced in Section 3. Finally, we briefly outline the current status and future perspectives for CGM systems in Section 4.

## 2. Mini-Invasive Biosensors for CGM

Dynamic glucose detection methods usually employ a tiny sensor inserted beneath the skin and maintained for a certain period of time in contact with the interstitial fluid. Most minimally-invasive systems for CGM are enzyme-based, whereas others are enzyme-free. For both enzyme and non-enzyme methods, numerous efforts have been focused on the preparation, functionalization and modification of the required electrodes [16,20,21].

### 2.1. Sensing Design and New Materials

Enzymatic electrodes use enzymes to catalyze reduction–oxidation (redox) reactions, and the movement of electrons could thus produce a concentration-dependent current or voltage that can be measured by electrodes. Frequently utilized electrodes for invasive sensor technology include microdialysis, micropores, microneedles, subcutaneous amperometric electrodes, and intravenous implantable devices. Currently, only microdialysis and subcutaneous devices have reached commercialization. Microdialysis systems utilize a hollow microdialysis fiber which is perfused with isotonic fluid from an ex vivo reservoir, and meanwhile IG freely diffuses into the fiber, and then is pumped to an enzyme-based electrochemical sensor. This system has smaller dimensions for implantation convenience, and the sensor is ex vivo, thus avoiding biofouling problems [22]. For subcutaneous methods, the design of the electrodes mainly aims at increasing the sensitivity, selectivity and biocompatibility.

A major historical advance in the in vivo application of glucose biosensors was the classic needle-type sensor, which generally consists of a platinum-iridium (Pt-Ir, φ 0.125 mm, Pt:Ir = 9:1)) wire working electrode with an immobilized mediator and enzyme on the surface, and/or a polymer coating for subcutaneous implantation. A silver/silver chloride (Ag/AgCl) wire is wrapped around the working electrode that serves as a counter electrode. Such devices are designed for human implantation sensors that continuously operate for a few days and be replaced by the patient. Algorithm correction for the transient differences (lag-time) between BG and IG concentrations have been investigated for future applications. Kumetrix (Union City, CA, USA) has proposed a hand-held, battery-powered electronic monitor with silicon micro-needles that are similar in size to a human hair. The aim is to develop a meter system that accepts a cartridge loaded with disposable sampling devices. This action will cause the micro-needle to penetrate the skin and draw a very small volume of blood (less than 100 nL).

Coil-type oxidase biosensors were developed based on the design of needle-type ones. Pt-Ir wire was wound up along a 30-gauge needle to form a coil-shaped cylinder, which had a length of ca. 1 mm with an outer diameter of 0.55 mm and an inner diameter of 0.3 mm. Such a design dramatically increased the sensing area of flexible implantable biosensors within an acceptable length range and created a ca. 0.07 mm three inner storage chamber for extra enzyme immobilization. With modifications such as etching of the surface of coil, hydrogel layer coatings and loading with certain drugs, the constant response of the sensor could be maintained for at least 60 days [23].

As for commercialized glucose sensors, Abbott (Chicago, IL, USA) has developed a series of BG monitoring products named FreeStyle since June 2000. A recent FreeStyle Libre device allows patients to measure glucose within 1 s by scanning the sensor with a reader, and the sensor could work for 14 days without calibration. As a result, the pain associated with testing is greatly reduced. In 2006, the Dexcom (San Diego, CA, USA) SEVEN, the first real-time continuous glucose monitoring system, was approved by the FDA. Recently the Dexcom G5, the first fully mobile CGM system, was launched. Dynamic glucose data could be accessed safely by a compatible smart device. Another transdermal implantable CGM system, the CT-100 (POCTech, Huzhou, China), utilizes a “parallel implant method”, which offsets the limitation of the length of the sensor by taking full advantage of the subcutaneous tissue and thus differs from Dexcom’s products. Such a design improves the sensitivity while preventing misoperation causing the sensor to go into the muscle tissue.

The diagnosis and management of diabetes mellitus require a tight monitoring of glucose concentration. An ideal sensor would be one that provides reliable real-time continuous monitoring of glucose variations throughout the day with high selectivity and speed over extended periods under harsh conditions. New sensing concepts, coupled with new materials and numerous technological innovations have been made for enhancing the capabilities and improving the reliability of glucose measuring devices.

The development of carbon nanotubes (CNTs) and graphene-based electrodes have been intensively studied, and they show good safety when used in implantable electrodes for in vitro testing [16]. With similar dimensions as redox proteins, carbon nanomaterials could be utilized as effective electrical wiring/connectors with redox enzymes, which is one of the most promising materials for enzymatic glucose biosensors. For bio-sensing applications, graphene and CNTs demonstrated higher sensitivity and faster response time than traditional electrodes at extremely low working potentials. Our group has carried out a comprehensive study of CVD-synthesized CNT fibers used as sensing electrodes to detect glucose solutions. The CNT fiber resembles an electric wire, relying on nanoscale surface topography and porosity, which can facilitate molecular-scale interactions with agents like enzymes to efficiently capture and promote electron transfer reactions [24,25]. A scalable synthesis of a multifunctional conducting polyacrylic acid (PAA) hydrogel for glucose detection that integrates a multifunctional matrix that includes reduced graphene and lutetium phthalocyanine was reported. This biosensor provided high sensitivity for the detection of glucose with a low detection limit [26]. However, better control of the physical and chemical properties is still needed for the fabrication of carbon nanomaterial-based biosensors, such as the miniaturization of the sensor, in vivo stability, separation processes for different type of CNTs, etc. Other non-enzymatic sensors, such as metallic oxide and strontium palladium perovskite also show enhanced sensitivity and improved detection limits, and thus avoid the need for expensive enzymes in the system [27,28].

**Figure 1:**
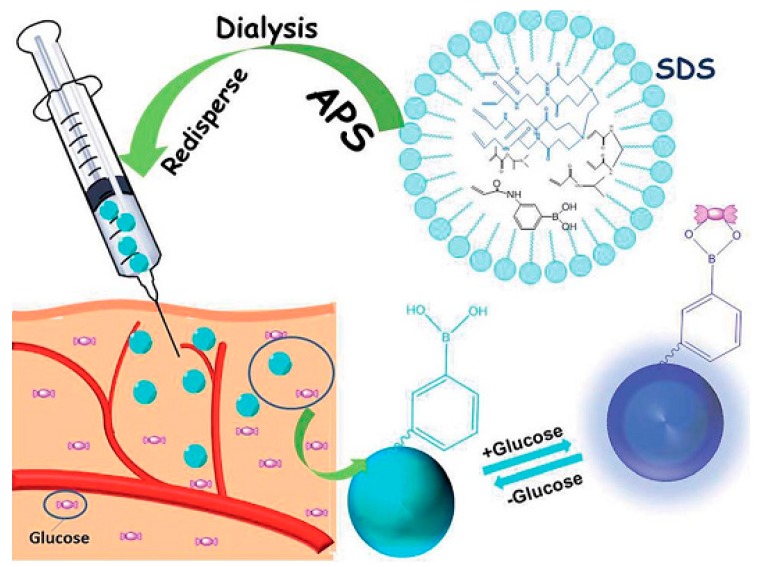
The schematic illustration of G1.0 PAMAM-functionalized microgels that can recognize glucose and emit blue fluorescence after injection. Reprinted with permission from [36].

Microgels, glucose-responsive polymer gels, have several advantages as glucose sensing systems due to their porous structures for carrying drugs, semi-solid properties for easy engineering into various shapes (beads, films, fibers, etc.), and potential biocompatibility for implantation. Since the first preparation of boronic acid-functionalized glucose sensitive hydrogels [29], many polymer gel-based glucose sensors have been developed based on the measurements of swelling pressure [30], fluorescence intensity [31], changes in reflection holograms [32,33] and diffraction of photonic colloidal crystal arrays [34]. Li et al. recently developed a series of inorganic-polymer hybrid microgels for optical glucose sensing, based on the immobilization of fluorescent quantum dots (QDs) or metal nanoparticles (NPs) into phenylboronic acid (PBA)-functionalized microgels [35]. Zhang et al. constructed an advanced glucose-sensitive platform on the basis of G1.0 PAMAM-functionalized microgels [36]. The beauty of this smart microgel, which differentiates it from other examples of traditional glucose sensors, is that it provides minimally invasive implantation and a fluorescent signal by transdermal transmission without any external links or electric power sources for CGM. A schematic illustration of G1.0 PAMAM-functionalized microgels that can recognize glucose and emit blue fluorescence after injection, is depicted in Figure 1.

**Figure 2:**
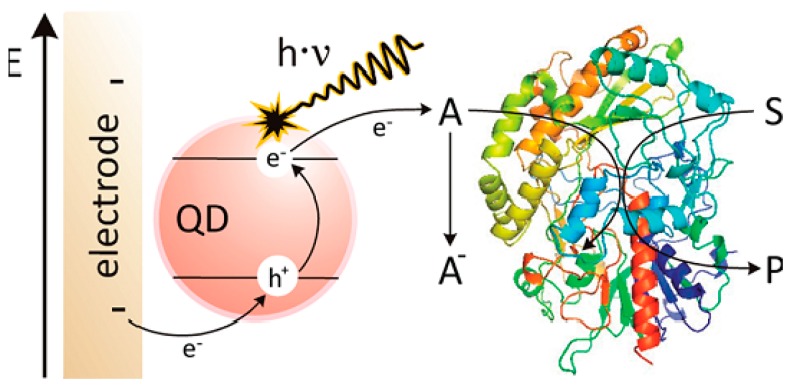
Illustration of the electron transfer steps after illumination of the QD electrode. Reprinted with permission from [40].

Semiconductor quantum dots (QDs), as photoluminescent nanomaterials, have been intensively studied in sensing and cell imaging due to their excellent performance and size-tunable optical properties [37]. Glucose could be detected by indirect measurements using photoelectrochemical QD sensors if combined with suitable enzymes [38,39]. Tanne et al. reported the indirect sensitive detection of glucose by creating a signal chain from glucose via glucose oxidase and molecular oxygen via CdSe/ZnS QDs toward the electrode [40], as shown in Figure 2. On the basis of the influence that the oxygen concentration has on the photocurrent, the enzymatic activity of glucose oxidase catalyzing the oxidation of glucose by the reduction of O_2_ was evaluated. During illumination, the photocurrent was reduced as a result of the oxygen consumption. The sensing properties of this type of electrode were strongly influenced by the amount of enzyme on top of the QD layer, which was found to be easily adjustable using the layer-by-layer technique. The aforementioned glucose sensors are based on signal chains of glucose-glucose oxidase-oxygen-QDs. However, the enzyme can also be coupled to an electrode by means of a shuttle molecule, which facilitates a mediated electron transfer from the biocatalyst. Zheng et al. constructed a photoelectrochemical electrode by alternately depositing water-soluble CdSe-CdS QDs and a mixture of [Co(phen)_3_]^2+/3+^ and poly(ethyleneimine) on a TiO_2_ electrode [41]. In this setup, the electrode was able to transfer charge carriers from the reduced enzyme, so that the obtained photocurrent depended on the concentration of glucose.

Nowadays, label free and real time optical detection methods are the most promising techniques in glucose monitoring. Complementary metal oxide semiconductor (CMOS) image sensore are becoming powerful tools for biosensing applications with the advantages of high signal to noise ratio, low power consumption, miniaturization and availability on smartphones and even easier to incorporate into compact medical diagnostic devices. In addition, CMOS image sensors can employ various glucose monitoring methods, including continuous glucose monitoring methods [42,43], enzymatic glucose monitoring methods [44,45], and thermal-based glucose monitoring [46], to operate at the in vitro and in vivo level.

**Figure 3:**
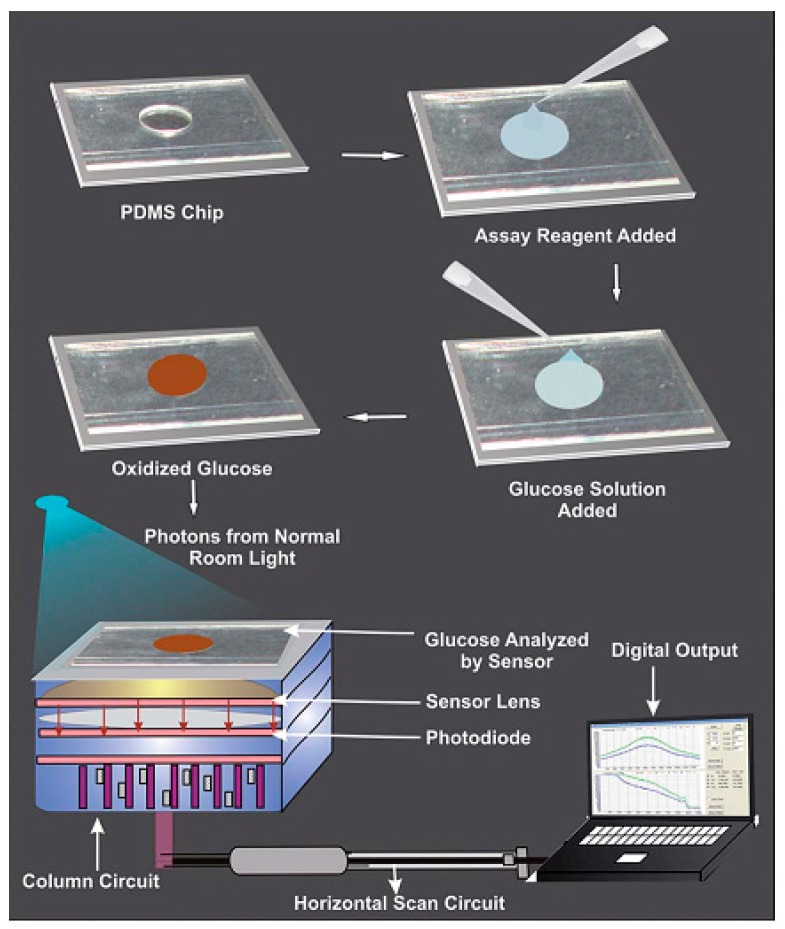
Schematic of PDMS chip utilization for monitoring of glucose solutions by a CMOS image sensor. Reprinted with permission from [44].

Kim et al. exploited a CMOS image sensor for detecting glucose levels by simple photon count variation with high sensitivity [44], as shown in Figure 3. Various concentrations of glucose (100 mg/dL to 1000 mg/dL) were added onto a simple polydimethylsiloxane (PDMS) chip and the oxidation of glucose was catalyzed with the aid of an enzymatic reaction. Oxidized glucose produces a brown color with the help of a chromogen during the enzymatic reaction and the color density varies with the glucose concentration. Photons pass through the PDMS chip with varying color density and hit the sensor surface. Photon count was recognized by the CMOS image sensor depending on the color density with respect to the glucose concentration and it was converted into digital form. By correlating the obtained digital results with glucose concentration it is possible to measure a wide range of BG levels with great linearity based on the CMOS image sensor and therefore this technique could promote a convenient point-of-care diagnosis method.

### 2.2. Novel Biocompatible Coatings for Sensors

The major challenge in the design of tissue-contacting materials for both bioaffinity and medical implantation sensors is to endure protein adsorption between surfaces and interfaces. Most minimally-invasive biosensors are surrounded by protective coatings that can strongly interact with proteins, thereby minimizing tissue reactions induced by device implantation [47]. To achieve a protein-resistant surface, the structure design of the anti-fouling material should be hydrophilic, electrically neutral, and contain hydrogen bond acceptors instead of donors. These requirements guide the design of anti-fouling formulations for sensing applications with novel structures that have non-specific protein resistance [48]. A considerable number of effective candidates have been identified, including organic/inorganic composites and biofunctional polymer architectures. Sol-gel derived silicates have been demonstrated to be highly compatible with enzymes [49]. Silica-based hybrid materials exhibit a fair biocompatibility both in vitro and in vivo, and are shown to be non-toxic and biocompatible coatings for glucose oxidase-based sensors. Stable glucose responses were obtained for the silica-polymer coated sensors both in buffered solutions containing bovine serum albumin and in serum [50,51]. Unfortunately, the anti-fouling properties of such systems were not discussed.

Various macromolecules, including natural, semi-synthetic, and synthetic materials are currently utilized in the fabrication of implantable CGM sensor coatings (cf. Table 1). Naturally occurring materials such as alginate, chitin, and chitosan and their derivatives are widely investigated for biosensors in the form of sheets, membranes and coatings, which offer the advantage of being comparable to the natural precursors and the biological environment is prepared to recognize and deal with their metabolites [52,53]. On the other hand, modified synthetic polymers with tailored structures and coating properties are often superior to traditional macromolecules, especially for their reduced immunogenicity. Commonly used biocomparable and biodegradable polymers includes poly(lactic co-glycolic acid) (PLGA), poly(ethylene glycol) (PEG), poly(hydroxyethyl methacrylate) (PHEMA), and poly(vinyl alcohol) (PVA), etc. [54]. Among the abovementioned polymers, HEMA-based materials constitute the first generation of efficient protein-resistant materials while PEG-derived materials represent the second generation [55]. Currently, they are extensively utilized in various areas and can be used to modify mini-invasive devices by the coating method. They are also very popular as hydrogel matrices, either alone or combined with other polymers [56].

Hydrogels are hydrophilic, water-insoluble three-dimensional polymeric networks represented as semi-open structures comprising entangled chains, which adsorb and store large amounts of water and are highly permeable to small molecules. The use of hydrogel coatings allows the diffusion of glucose through the swollen hydrogel layer. The degree of glucose diffusion could be readily modulated by physically/chemically control of the cross-linking density of the hydrogel, which consequently affects the water content and mechanical strength of polymer network. Both in vitro and in vivo animal tests have demonstrated better performance with biocompatible polymer hydrogel outer layers for a mini-invasive needle-type glucose sensor.

Despite oxidation problems that could alter the anti-fouling properties during long-term usage, PEG-derived anti-fouling matrices remain popular and have been widely investigated [57]. Similar to PEG, poly(ethylene glycol) methyl ether methacrylate (PEGMA) and PEGMA-analogous hydrogels were also investigated for the design of the next generation of anti-biofouling materials [58]. The results revealed that the molecular weight of the PEGMA homopolymer directly affected the overall hydrophilicity of the gel, which could be attributed to both the intermediate hydrophilic chain length and intermediate crosslinking degree. Furthermore, the results of micro-biofouling by bacteria and blood cells demonstrated that PEGMA hydrogels offered the perfect non-fouling ability, showing application potential for the coating of devices in contact with blood and the in vivo controlled release of drugs from nonfouling hydrogels.

### 2.3. Drug Deliver and Its System

**Figure 4:**
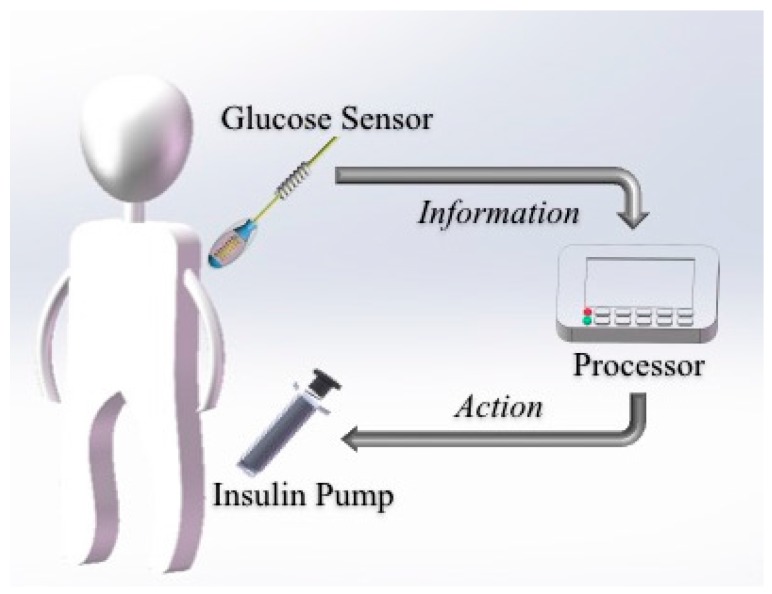
Illustration of closed-loop glycemic management system utilizing the ‘Sense and Act’ method for optimized insulin delivery.

Drug delivery is a potential field of application for CGM devices [59]. In recent years, architectures for sustained and controlled delivery of drugs has been extensively studied due to several problems in common drug delivery systems, such as limited stability, inopportune leakage and poor solubility, especially for CGM systems. Modern closed-loop BG controlling systems have been developed by various organizations around the world, by merging glucose-sensing devices and insulin-delivery carriers for optimized dosage of insulin according to detected glycemic levels (Figure 4). Such BG management systems utilize a ‘Sense and Act’ feedback-loop design to realize appropriate corrective action by timely and optimal dosing. The pump dispenses the right amount of insulin as the sensor relays the information. The development of these responsive drug delivery systems is anticipated to dramatically change diabetes management and patient monitoring. It is expected that the release of different drugs could be controlled by a microprocessor in response to the signal from a biosensor via different release kinetics.

Based on the biocoating, intelligent drug releasing functionality was also investigated to make the system more integrated, and ensure the long-term usage of the device [60]. For instance, a PLGA-copolymer, PVA and PEG combined system which has been shown to achieve a continuous release of dexamethasone for up to one month that decreases extravasation of leukocytes, and inhibits production of proteolytic enzymes and chemoattractive factors was already discussed [61,62]. A masitinib-releasing PLGA carrier delivers locally from soluble PEG-coated CGM sensors were developed to reduce the host-implant response. The releasing action could be attributed to the CGM sensor output fluctuations over time, which was monitored for 21 days. The results showed relatively improved performance in terms of sensing ability in the drug-delivery implant group compared to the control group [63].

Recently, a novel implantable electrochemical sensor was fabricated by utilizing a spiral Pt-Ir alloy electrode to increase the active area of the working electrode as well as enhance the loading of glucose oxidase on the biosensor. The result showed that the sensitivity of the as-prepared spiral sensor was 20 nA/(mmol/L)~30 nA/(mmol/L), which provided a linear detection of physiological glucose concentrations between 2 and 30 mmol/L. The sensors also showed good repeatability, selectivity, and stability, which could be tuned by adjusting the amount of the polyurethane (PU) semi-permeable membrane coating [64]. More recently, the PU coating was further modified by a PVA-PEG hydrogel that enabled zero-ordered release of dexamethasone, and the release duration was controlled as a function of film gelation density. The cumulative released amount could be adjusted by changes in the molecular weight of the PEG. As a matter of fact, PEG derivatives were revealed to be effective candidates for sensor coatings as well as controlled release of supposed drugs [58,65].

## 3. Non-Invasive Technology

Non-invasive techniques have received significant research interest due to the highly sensitivity and better patient compliance, contrary to invasive ones. Typical non-invasive biosensors based on different approaches include iontophoretic extraction of glucose through the skin, surface plasmon resonance, Raman spectroscopy in aqueous humor, visible or near-infrared (NIR) spectroscopy, polarimetry, photo-acoustic probes, and fluorescence methods. Although results from these innovative devices require frequent calibration against direct BG data, they might be an alternative choice for future continuous glucose measurement. Consequently, the challenge of preparing accurate level sensors to biomonitor conveniently and painlessly BG information remains to be solved.

### 3.1. Tear Sensing Designs

Non-invasive, contact lenses are one of the most appealing ways to achieve constant contact with tear fluid and monitor glucose in tears. Such a method could be used for normal conditions as well as for clinical trials. During the course of treatment, the glucose could be continuously monitored. Contact lenses have to meet a certain number of criteria, and should be made of soft, comfortable materials with constant mechanical properties such as hydrogels or plasticized polymers that could withstand the eye movements and blinking. Moreover, the lenses also should be non-toxic and to allow for a certain amount of oxygen and tear fluid to pass through to the eye. Some interactions between contact lenses and the tear film were discussed. The presence of the contact lens immediately provides the potential for both biophysical and biochemical phenomena. The biophysical phenomena resulting from lens-tear interactions are often directly observable as the increased osmotic pressure caused by enhanced evaporation of the tear film. The biochemical outcomes revealed the difference in electrolyte concentration between the anterior and posterior tear films [66,67].

**Figure 5:**
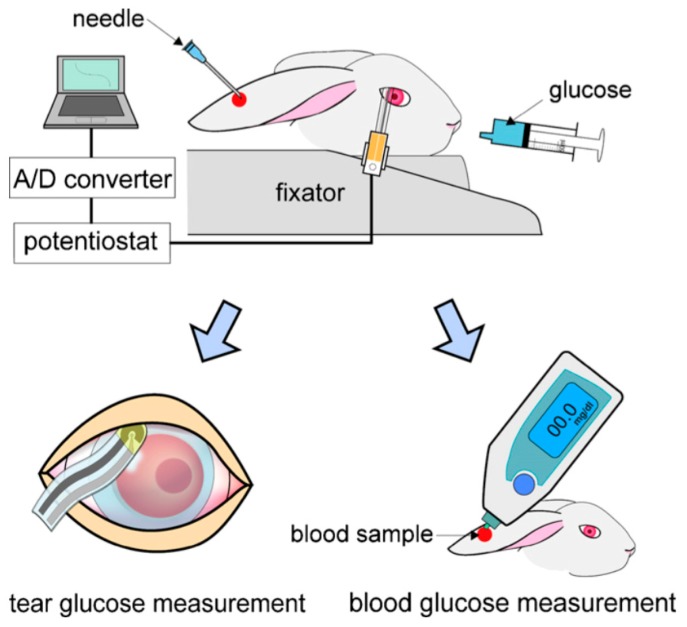
Measurement method of tear glucose concentration with a contact lens biosensor. BG levels were simultaneously measured by a commercial BG monitoring kit. Reprinted with permission from [69].

Several types of sensors have emerged in recent years based on different monitoring methods, including electrochemical and spectral determination. Electrochemical sensors have become more complicated and miniaturized at the same time. A protocol consisting of a soft polymer lens covered by an enzyme-based sensor was reported, using a tiny metal electrode laid out of the lens to detect electrochemical signals in response to glucose [68]. Another design included integration of an electrical circuit, a sensing probe, a body-wired communication transmitter, a power supply, and a PDMS contact lens by using MEMS techniques. During the monitoring, the glucose solution was orally administrated to rabbits and the BG level was measured using a glucose monitoring kit (Figure 5). This biosensor showed rapid responses to glucose and an appropriate calibration range (0.03–5.0 mM), which covered the reported tear glucose levels [69].

**Figure 6:**
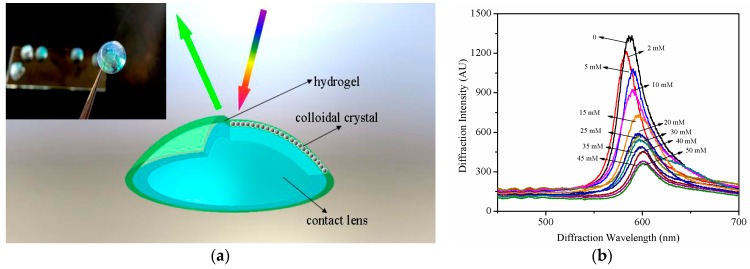
(**a**) Diagram and photograph (insert) of a physical hydrogel photonic crystal sensing lens; (**b**) Diffraction wavelength shifts with the variation of the glucose concentration in artificial tear solution.

Spectral sensors for CGM, on the other hand, were simplified. Contact glucose-sensing lenses based on hydrogels that contain phenylboronic acid (PBA) and photonic crystals that function without requiring power were proposed [70,71,72]. Such soft hydrogel lenses embedded an array of mono-disperse particles, which could diffract electromagnetic waves according to Bragg’s law. It is well known that PBA-modified hydrogels are able to bind *cis*-diols in glucose, leading to volumetric changes and forcing the lens to expel water, altering the array spacing, thus changing the perceived color of the lens. Moreover, the color changes caused by the reaction are quick and reversible. Other photonic crystal embedded lenses were developed to improve the sensitivity or use different detection spectrometers [73,74]. Recently, a plastic contact lens outfitted with a physically gelated photonic crystal hydrogel was synthesized for CGM in our group. The hydrogel was physically crosslinked from PVA-PEG system, which could offer an anti-fouling layer for the penetration of glucose. This hydrogel was subsequently modified with 4-boronobenzaldehyde, inproving the sensitivity for glucose, and a prototype of such a lens is shown in Figure 6a. The sensing behavior of the lens was monitored by a reflection photospectrometer, and the results showed that the diffracted wavelength was relatively shifted with the increasing glucose concentration in artificial tear solution (Figure 6b). Despite the exciting design, it remains difficult to measure glucose concentrations in tears using contact lenses. Because of the very low amount of glucose present in healthy control subjects (3.59 mM) and for diabetic subjects (4.69 mM), the tear glucose levels measured appear to vary with the volume of the aqueous tear fraction collected.

### 3.2. Salivary Biosensors

Many authors have found higher glucose salivary levels in diabetic patients than in non-diabetics [75,76,77,78]. Such investigations mainly aimed at exploring whether diabetic control could be monitored by a non-invasive salivary glucose measurement method. Saliva is a great diagnostic fluid providing an alternative to direct blood analysis via the permeation of blood constituents without any skin-piercing for blood sampling [79]. Actually, saliva glucose concentrations range approximately from 20 to 200 mmol/L in normal and diabetic individuals, and closely follow circadian BG fluctuations [80]. Saliva and BG levels correlate reasonably well in a sample of individuals [81,82,83], however, a much stronger correlation is observed within the same individual, enabling BG concentrations to be estimated from saliva glucose measurements [84].

Various attempts have been made by researchers to develop glucose monitoring systems for salivary measurements. Lambert et al. used conventional measurement through colorimetric assays [85]. Lipson et al. detected the saliva glucose level through NIR/Raman-based spectroscopic techniques [86], and Yamaguchi et al. used Clarke-type electrode-based amperometric measurements, etc. [80]. Taking clues from these developments, Soni et al. developed an optical biosensor for direct determination of salivary glucose by using glucose oxidase enzyme immobilized on filter paper strips (specific activity 1.4 U/strip) and then reacting it with synthetic glucose samples in the presence of a co-immobilized color pH indicator [83]. The filter paper changed color based on the concentration of glucose in the reaction medium and hence, by scanning this color change (using RGB profiling) through an office scanner and using open source image processing software (GIMP), the concentration of glucose in the reaction medium could be deduced.

**Figure 7:**
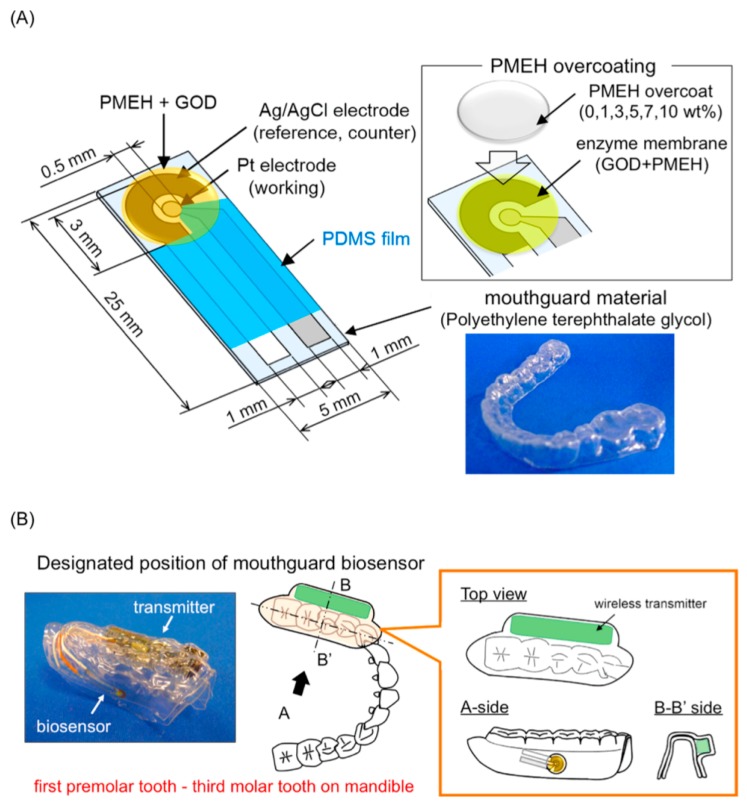
(**A**) Schematic image of the glucose biosensor on the polyethylene terephthalate glycol mouthguard support. Pt and Ag electrodes were formed on the PETG through a sputtering process. Each electrode sensor consisted of a 0.20 mm^2^ Pt working electrode and a 4.0 mm^2^ Ag/AgCl reference/counter electrode, both insulated with PDMS on a 0.5 mm thick PETG layer. 30 units of GOD were applied to the sensing region of the working electrode. In order to optimize enzyme entrapment, 2.0 mL of 1.0 wt% PMEH solution was spread over the sensing region to form the PMEH overcoat; (**B**) Schematic image of the mouth-guard biosensor custom-fit to the patient’s dentition. The device consists of a glucose sensor and wireless transmitter incorporating a potentiostat for stable glucose measurement. The sensor was designed to fit the mandibular dentition from the first premolar up to the third molar. The wireless transmitter was neatly encased in PETG. Reprinted with permission from [94].

Wearable sensors have recently attracted considerable interest owing to their promise for real-time monitoring of the wearer’s health and fitness in a wide range of biomedical, sport and military scenarios [87,88]. Recent efforts have led to wearable biosensors for detecting chemical biomarkers in human fluids that can be obtained non-invasively, e.g., tears, sweat or saliva [89,90,91,92]. Kim et al. reported a mouth-guard biosensor for continuous monitoring of salivary lactate and other chemical components [79,93]. A mouth-guard glucose sensor produced using micro electromechanical systems (MEMS) techniques would offer promise as a minimally-invasive, painless, continuous, custom-fitted and wireless solution for self-monitoring of glucose. Mitsubayashi et al. have developed a detachable “Cavitas sensor” used in the human oral cavity for non-invasive monitoring of saliva glucose [94], as shown in Figure 7. The mouth-guard glucose sensor consisted of a platinum and silver/silver chloride electrode, with glucose oxidase immobilized by entrapment with poly(MPC-co-EHMA) (PMEH), on a custom-fitted monolithic mouth-guard support with a wireless transmitter, thereby enabling telemetric measurement of saliva glucose.

### 3.3. Other Methods

Recently continuous glucose monitoring devices have become available and could provide more detailed data on glucose excursions. In future applications such continuous glucose sensors may become a critical component of closed loop insulin delivery systems and, as such, must be selective, rapid, predictable and acceptable for continuous patient use. Many potential sensing modalities are being pursued for this, including optical techniques.

Optical sensors use light of variable frequencies to detect glucose, utilizing different interaction properties of light with glucose molecules in a concentration-dependent manner [95]. Compared to conventional glucose sensors, optical sensors benefit from the absence of electromagnetic interference, simple design and handling as well as low cost. Miniaturization of optical sensors is easily achievable without compromising performance and dramatically reduces repulsion reactions. Evidently, optical sensing technology can provide an interesting alternative to more established electrochemical sensors and will contribute to better flexibility concerning the design of CGM systems, their cost, materials used, etc. [96]. Based on this optical transducing technology, numerous optics-based CGM systems have been developed, such as fluorescence, infrared absorption spectroscopy, and Raman spectroscopy.

Fluorescence uses the principle of varying light emission from molecules in different states. It is fast, reagentless and extremely sensitive. Many fluorescence-based glucose sensors are based upon the affinity sensor principle where glucose and a fluorescein-labelled analogue bind competitively with a receptor site specific for both ligands [97,98]. Other fluorescence techniques use enzyme- catalysed reactions to change fluorescent status. Fluorescein may be bound to glucose oxidase, allowing energy transfer between the flavin group of GOx and the fluorescein. The GOx enzyme may be further utilized by measuring oxygen consumption or hydrogen peroxide production by fluorescence [99,100]. GOx can also be used as glucose-binding protein, utilizing its intrinsic fluorescent properties and may be used without its flavin group with a fluorescent label [101].

Near infrared (NIR) light (wavelength 0.7–1.4 μm) provides an optical window in which 90%–95% of light passes through the stratum corneum and epidermis into the subcutaneous space independent of skin pigmentation. It is already successfully used to non-invasively monitor the concentration of oxygenated and deoxygenated haemoglobin in the preterm infant brain [102]. BG has been measured across the oral mucosa using NIR [103]. The oral mucosa is an obvious candidate for sensing, as it is well vascularized and may be trans-illuminated. However, any oral measurement includes saliva, which may contain a different glucose concentration, and residual food may contain interferents. NIR spectroscopy has been measured across the tongue [104]. The standard error of prediction was 3.5 mmol/L, with significant variability caused by variability in tissue fat. NIR therefore ideally requires consistent tissue fat in addition to a path length in the order of 5 mm [105]. In order to solve some of the problems posed by transdermal optical techniques, NIR has been proposed in combination with techniques for sampling ISF, including sonophoresis [106].

Raman spectroscopy assesses scattering of single wavelength light. This is dependent on rotational or vibrational energy states within a molecule and highly specific absorption bands are seen with Raman spectroscopy which can be used to identify and quantify molecules. It has the benefit of reduced interference from water compared with MIR or NIR spectroscopy. However, the Raman signal is weaker than that of other technologies, requiring powerful detectors for physiological concentrations of glucose. The Raman signal is also susceptible to turbidity, haematocrit, skin thickness and melanin. Sufficient sensitivity can be achieved by surface-enhanced Raman spectroscopy (SERS). The electromagnetic mechanism of SERS is that when an electromagnetic wave interacts with a metal surface, the fields at the surface are different than those observed in the far field. If the surface is rough, the wave may excite localized surface plasmons on the surface, resulting in amplification of the electromagnetic fields near the surface. If one assumes that there is enhancement of the intensity of the incident and scattered fields (albeit at different wavelengths), then the possibility of a large enhancement of Raman scattering intensity arises [107]. And now some reports utilized the SERS signal probes served as the molecular recognition agent in response to the concentration of glucose. Torul et al. fabricated a SERS substrate by modifying two component self-assembled monolayers (SAMS) on the surface of gold nanorod particles [108]. The variation of the SERS signal of SAMS showed the response to the concentration of glucose, and a low detection limit of 0.5 mM was obtained. Kong et al. proposed a novel glucose binding mechanism by using phenylboronic acid as the receptor for saccharide and forming a glucose-alkyne-boronic acid complex on SERS substrate, which exhibited a new Raman peak at 1996 cm^−1^ [109]. Thus, the novel technology offered a high sensitivity for SERS glucose sensing.

## 4. Future Perspectives

The worldwide increase in the number of diabetic patients has encouraged scientists to put great efforts into the field of continuous glucose monitoring (CGM) devices, which would dramatically alter the treatment of diabetes as well as the life quality of diabetic patients. So far, there are only a few commercial CGM devices, developed by Medtronic (Minneapolis, MN, USA) and Dexcom, which have been approved by the FDA and their current lifetimes are less than one week. No commercial CGM devices are available for long-term monitoring, however, it is extremely important for T1D, which accounts for around 10% of diabetic patients. The existing studies of long-term mini-invasive biosensors remain challenging, mainly due to the foreign body responses such as biofouling, fibrous capsule and inflammation. To overcome foreign body responses and improve the life-time of implantable biosensors, we believe the following issues should be addressed: (1) improving the loading amount and stability of enzymes. The coil type electrode design is one way to load more enzyme to reduce the effects from accumulation of H_2_O_2_, glucose oxidase molecular cloning or encapsulation in silica sol-gel may be another way to reduce the degradation of cross-linked enzymes [110]; (2) a number of interferences (e.g., acetaminophen, ascorbic acid, and uric acid) can affect sensor response as they are electroactive during the oxidation of hydrogen peroxide, although semi-permeable membranes with designed porosity, like polyurethane, are able to alleviate this effect, At the meantime, decreasing the work potential through electrode design is another route to reduce interference; (3) to reduce foreign body responses once the biosensors are implanted, the synthesis of anti-fouling films is a common strategy to improve glucose sensor functionality against the initial adhesion of proteins. The introducing of biocompatible hydrogel coatings or collagen encapsulation for sensing devices are other key approaches to improve the lifetime. The use of small anti-inflammatory and/or antigenic molecules, such as dexamethasone, to influence tissue integration has been intensively studied. Here, the drug delivery system with biocompatible and controllable particles, like poly(lactic-co-glycolic acid) is pursued to limit inflammation and take advantage of the wound healing properties of this material.

Despite the fact that the results from non-invasive devices require frequent calibration against invasive ones, the innovative design might be an alternative choice for glucose measurements than traditional implanted electrochemical biosensors. Two possible techniques have the most chance for non-invasive glucose monitoring, one is tear glucose sensors and another is salivary biosensors. Detecting glucose in tear fluid has been established for over twenty years, and the major challenge for traditional contact lens biosensors is that the amount tear fluid is not high enough to cover the electrode to construct an electrochemical cell, and this thus leads to less accuracy in this type of sensor. Compared with electrochemical sensors, a plastic contact lens outfitted with a physically gelated photonic crystal hydrogel maybe a possible route for CGM in the future. A salivary biosensor incorporating Pt and Ag/AgCl electrodes on a mouth-guard support as dental material with an enzyme membrane was developed and tested by Mitsubayashi’s group [94], and this invisible mouth-guard biosensor integrated with a low energy wireless module could be an ideal candidate for future real-time monitoring of salivary glucose levels.

Non-enzymatic sensor show enhanced sensitivity and detection limits, while avoiding the expensive and fragile enzymes in the system, and thus it is also a promising choice for the next generation of CGM. Although there are large number of research work on this area, the clinic products are not yet available in the market. The ultimate goal for treating diabetic patients is to construct an artificial pancreas, combining CGM and insulin dosage system, the life time of CGM is the greatest challenge, and are required for deeply and comprehensively studied in the future.

## Figures and Tables

**Table 1:** Biochemical aspects of commonly used polymers.

Polymer	Characters	Formation	Applications
**Natural**			
Alginate	Immobilization of glucose oxidase	Hydrogel and membrane	Drug delivery
Collagen	Extracellular matrix component	Hydrogel, membrane and sponge	Scaffolds
**Semisynthetic**			
Chitin, Chitosan		Hydrogel, membrane and fiber	Anti-microbial and drug delivery
**Synthetic**			
PLGA	Negligible protein adsorption	Micelle and hydrogel	Coating, drug delivery and scaffolds
PHEMA	Negligible protein adsorption	Hydrogel	Coating
PVA	Geltaion and mechanical properties	Hydrogel, membrane and sponge	Coating and drug delivery
PEG	Negligible protein adsorption	Hydrogel and membrane	Coating and drug delivery
PEGMA	Negligible protein adsorption	Hydrogel	Coating
